# Direct Air Capture of CO_2_ through Carbonate Alkalinity Generated by Phytoplankton Nitrate Assimilation

**DOI:** 10.3390/ijerph20010550

**Published:** 2022-12-29

**Authors:** Jing Su, Hui (Henry) Teng, Xiang Wan, Jianchao Zhang, Cong-Qiang Liu

**Affiliations:** 1 School of Earth System Science, Tianjin University, Tianjin 300072, China; 2 Key Laboratory of Horticultural Plant Biology, The Ministry of Education, College of Resources and Environment, Huazhong Agricultural University, Wuhan 430070, China; 3 Hubei Geological Survey, Wuhan 430034, China

**Keywords:** climate change, carbon removal strategies, CO_2_ sequestration, carbonate alkalinity, nitrate assimilation, direct air capture

## Abstract

Despite the consensus that keeping global temperature rise within 1.5 °C above pre-industrial level by 2100 reduces the chance for climate change to reach the point of no return, the newest Intergovernmental Panel on Climate Change (IPCC) report warns that the existing commitment of greenhouse gas emission reduction is only enough to contain the warming to 3–4 °C by 2100. The harsh reality not only calls for speedier deployment of existing CO_2_ reduction technologies but demands development of more cost-efficient carbon removal strategies. Here we report an ocean alkalinity-based CO_2_ sequestration scheme, taking advantage of proton consumption during nitrate assimilation by marine photosynthetic microbes, and the ensuing enhancement of seawater CO_2_ absorption. Benchtop experiments using a native marine phytoplankton community confirmed pH elevation from ~8.2 to ~10.2 in seawater, within 3–5 days of microbial culture in nitrate-containing media. The alkaline condition was able to sustain at continued nutrient supply but reverted to normalcy (pH ~8.2–8.4) once the biomass was removed. Measurements of δ^13^C in the dissolved inorganic carbon revealed a significant atmospheric CO_2_ contribution to the carbonate alkalinity in the experimental seawater, confirming the occurrence of direct carbon dioxide capture from the air. Thermodynamic calculation shows a theoretical carbon removal rate of ~0.13 mol CO_2_/L seawater, if the seawater pH is allowed to decrease from 10.2 to 8.2. A cost analysis (using a standard bioreactor wastewater treatment plant as a template for CO_2_ trapping, and a modified moving-bed biofilm reactor for nitrate recycling) indicated that a 1 Mt CO_2_/year operation is able to perform at a cost of ~$40/tCO_2_, 2.5–5.5 times cheaper than that offered by any of the currently available direct air capture technologies, and more in line with the price of $25–30/tCO_2_ suggested for rapid deployment of large-scale CCS systems.

## 1. Introduction

Global CO_2_ emission surged strongly in the past three years following a three-year lull, despite the deployment of renewable energies and the continuous increase in energy efficiency. The accelerating upswing rate, 2% in 2017 [1,2] and 2.7% in 2018 [3], and the projected 2019 increase of atmospheric CO_2_ (5.2 ± 0.9 Gt, or 2.5 ± 0.4 ppm) [4] only reinforces the notion that negative emission technologies (NETs) are necessities in the next 2 to 3 decades, if we are to curb the end-of-century warming to 1.5 °C, while continuing the reliance on fossil fuels for economic development. Current estimates show the total tonnage of CO_2_ needing to be removed through NETs in the 21st century amounts to 100–1000 billion [5]. This sizable quantity may herald the emergence of the NET industry in light of the recently enacted carbon tax credit in the US ($50 for a ton of CO_2_ buried and $35 a ton for CO_2_ re-utilized).

Available NETs are either biological (e.g., forestry and land management), chemical (e.g., direct air capture), or geological (e.g., mineral carbonation), depending upon the carbon removal mechanism. At present, most 1.5 °C pathways are land-plant based, that is, relying heavily on afforestation and crop plantation, followed by bioenergy generation with carbon capture and storage (BECCS). However, this scheme is severely fettered by land availability [6]. Recent progress in direct air capture (DAC) [7] brings a new and engineering perspective to this field. The current DAC technique uses a conventional approach (large mechanical fans driving ambient air through chemical adsorbents) to trap CO_2_, followed by heat-induced degassing to recycle the adsorbents. While operable at small scales, such apparatus raises serious questions concerning the feasibility (scalability, capital investment, and energy input) of having any significant effect on atmospheric CO_2_ reduction, in addition to the need for long term and safe storage of compressed CO_2_ [8].

Natural carbon management in the Earth system is attained by converting CO_2_ to aqueous species HCO_3_^−^ and CO_3_^2−^ and storing them as carbonate alkalinity in seawater. On geological scales, this scheme proceeds through weathering in the form of

CO_2_ + H_2_O + CaCO_3_ (calcite) = Ca^2+^ + 2HCO_3_^−^ (1)

for carbonate substrates or

2CO_2_ + H_2_O + CaSiO_3_ (wallostonite) = Ca^2+^ + 2HCO_3_^−^ + SiO_2_ (quartz) (2)

for silicate rocks. Such reactions remove 1 to 2 moles of atmospheric CO_2_ for every mole of minerals dissolved, and are responsible for sequestering 0.5 Gt CO_2_ annually [9]. The resultant seawater alkalinity is counterbalanced through carbonate mineral precipitation (marine biota formation), and ultimately transported to the deep by the biological pump.

A large amount of work has been devoted to developing CO_2_ removal technologies mimicking reactions such as Equations (1) and (2). To date, however, a breakthrough has yet to emerge to bring this approach close to the readiness level for carbon management [10]. On the one hand, the development is stymied technically by a bottleneck, resulting from the slow reaction kinetics commensurate only to geological time scale. On the other hand, even if the reaction rates can be artificially accelerated with minimal energy input, the magnitude and expenses of rock mining/CO_2_ transport and related processing are prohibitively too immense to be manageable financially and logistically. For example, an estimate shows that the amount of rocks needing to be weathered every year just to offset the world’s current level of new emissions is on the order of 100 billion tons [11]. As a comparison, the total US coal production from 1949 to 2011 is only about 46 billion tons.

Despite the lack of a leapfrogging advance, carbonate alkalinity based on CO_2_ trapping remains a rational option for climate mitigation because of the low risk to disturb earth system and the virtually unlimited natural carbon storage capacity. Nevertheless, radically different approaches need to be devised to deal with the sluggish reaction rates and the enormity of needed reactants (i.e., minerals). Here we present a different strategy, using microbial proton consumption, to circumvent rock weathering. The new scheme combines the efficiency and speed of microbial growth and the immense capacity of ocean and lithosphere to revitalize the seawater alkalinity approach and to broaden the NET portfolio.

## 2. Materials and Methods

### 2.1. Microbial Culture Experiments

Marine and freshwater phytoplankton communities were cultured under different nitrogen nutrient conditions. Seawater samples were first collected from the Bohai Bay in Tianjin city and a paddy field in Guangdong province, and subsequently cultured for microbial growth using the enriched seawater (ESAW) medium (modified after Harrison [12]) and the Wright Chu (WC) medium for freshwater (Supplementary Materials File S1). Dominant phyla in the samples were found to be cyanobacteria, green algae, and diatom. The culture was allowed to grow at 25 °C in a dark greenhouse illuminated by fluorescent lamps (2000 lux), with a 12-h:12-h on/off cycle.

To maintain an optimum nutrient level, 0.2 mL stock medium was added every three days. A sterile breathable membrane was used to seal the pool to ensure air circulation and prevent water loss through evaporation. The media were measured daily for pH and sampled every three days for C content determination.

### 2.2. Simples Analysis

Total Organic Carbon (TOC) and Total Carbon (TC) analyses of water samples were conducted using a total organic carbon analyzer (OI 1030W). For the TOC measurement, the filtered samples were pre-acidified with ~10% H_3_PO_4_ solution to remove inorganic carbon, while water samples for the TC measurement were filtered without pre-acidification. Dissolved inorganic carbon (DIC) was calculated by subtracting TOC from TC in water samples. Bio-carbon content of the microbial biomass (collected from biofilm on the substrate and rinsed by diluted or 10% HCl) was determined by an elemental analyzer (Vario EL III, Elementar, Germany). The carbon isotope measurement was performed by mass spectrometry (Finnigan MAT 253), with an analytical precision of ±0.1‰, and the results were expressed with reference to VPDB.

Biofilm formed in the experiments was fixed with 2% glutaraldehyde, dehydrated in concentrated ethanol, and gold-coated before imaging by SEM (Hitachi Su3500).

See SI material for a detailed description of the culture medium composition.

### 2.3. Working Principle

Inspecting the chemistry of Equations (1) and (2), one can readily see that the role of mineral weathering in these reactions is to neutralize the acidity generated by CO_2_ dissolution. A crucial aspect to note is that CO_2_ dissolution is not a kinetic hindrance for the purpose of alkalinity trapping because of CO_2_ hydration and the ensuing dissociation reactions

CO_2_ + H_2_O ⇔ H_2_CO_3_ ⇔ H^+^ + HCO_3_^−^ (3)

can reach equilibria in a matter of hours. It then follows that capturing CO_2_ in water is practically achievable, if a persistent alkaline condition can be generated and maintained in a timely fashion, to consume the protons produced in Equation (3).

The new approach we adopted to supplant mineral weathering is marine phytoplankton nitrogen assimilation. Nitrogen is a critical component of life. Other than diazotrophs that are able to directly utilize atmospheric N_2_ via biological nitrogen fixation (BNF), most organisms assimilate aqueous nitrate through enzyme (*nitrate and nitrite reductase*) catalysed processes, where NO_3_^−^ is first reduced to NO_2_^−^, which subsequently undergoes further reduction to form NH_3_. The overall reaction of nitrate assimilation can be written as

(4) $${{\mathrm{NO}}_{3}}^{-}+\mathrm{NADH}+9H^{+}+8e^{-}\overset{nitrate\;and\;nitrite\;reductase}{\to }\;{{\mathrm{NH}}_{3}}^{\;}+3H_{2}O+{\mathrm{NAD}}^{+}$$

The consumption of H^+^ in Equation (4) ultimately begets an alkaline environment over the course of phytoplankton growth [13] through

(5) $$106{\mathrm{CO}}_{2}+138H_{2}O+16{{\mathrm{NO}}_{3}}^{-}\;\overset{photosynthesis}{\to }\;({\mathrm{CH}}_{2}O{)}_{106}({\mathrm{NH}}_{3}{)}_{16}+16{\mathrm{OH}}^{-}+138O_{2}$$

Notice that ammonia assimilation, the conversion of ammonia to amino acids, will not produce this desired result because the overall reaction

106CO_2_ + 106H_2_O + 16NH_4_^+^ → (CH_2_O)_106_(NH_3_)_16_ + 16H^+^ + 106O_2_ (6)

brings about a net acidity increase.

## 3. Results and Discussions

### 3.1. Carbonate Alkalinity

**Figure 1:**
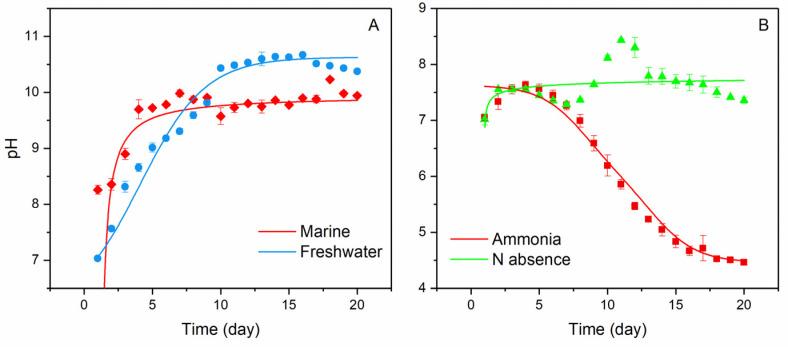
pH changes in different culture environments over 20 days of incubation, showing rising pH with time in nitrate condition for both marine and freshwater communities and (**A**) declining pH over time in ammonia condition as well as the relatively stable pH, when N is absent in freshwater (**B**). Error bars represent range of the measurements (n = 3).

**Figure 2:**
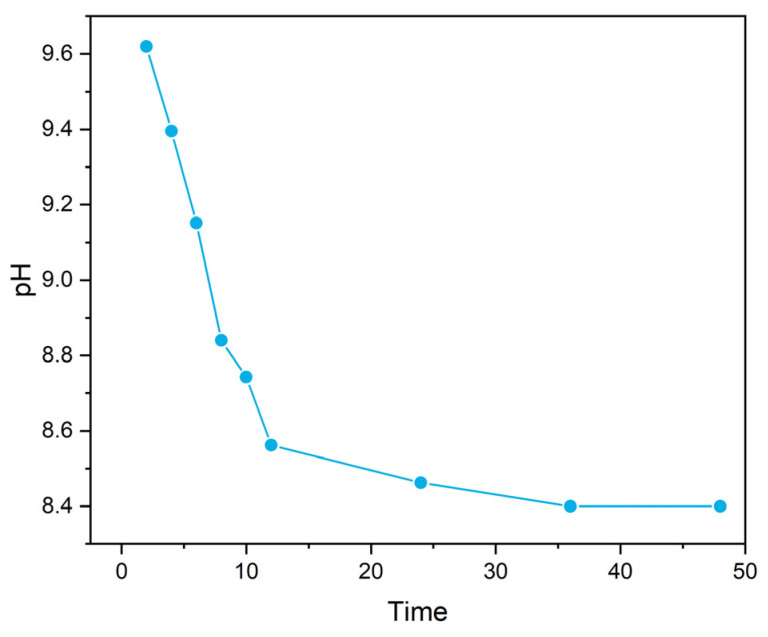
pH changes on time scale after continuous aeration.

**Figure 3:**
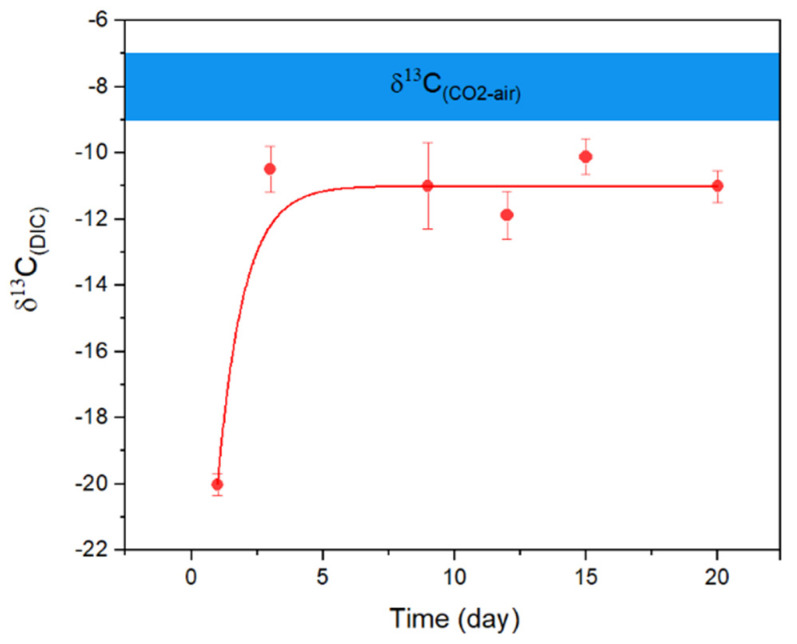
Evolution of δ^13^C_(DIC)_ in marine microbial culture over 20 days of incubation showing that the isotope composition approached the level of current atmospheric CO_2_ (blue bar at approximately −8‰). Error bars represent the range of the measurements (n = 3).

Consistent with Equation (5), both marine and freshwater microbial growth showed a persistent pH increase when nitrate was used as a N source (Figure 1A). The highest pH in seawater reached ~10.2 within 3–5 days, with total aqueous carbon (sum of biomass, organic, and inorganic carbon) increased by approximately 12 times. The alkaline conditions were able to maintain through the continued supply of nitrate. However, once the biomass was filtered out, the pH began to decrease, and ultimately reached a stable level of ~8.40 overnight (with aeration, Figure 2); meanwhile, the total dissolved inorganic carbon (DIC) in the media increased by ~80%, relative to the original seawater. Carbon stable isotope measurements showed that the δ^13^C_DIC_ values changed from the initial ~−20‰ to ~−11‰ within a 20-day incubation period, approaching the atmospheric value of ~−8‰ [14] (Figure 3) and signalling the occurrence of air CO_2_ absorption (Equation (3)), by seawater in the system. Under the same conditions, the freshwater microbial community was able to bring up the pH even higher (~10.5) within a similar time frame, with the total carbon increasing by ~23 times.

The pH-time relation shown in Figure 1 is expected from the biomass growth curve that increases exponentially during the logarithmic phase, followed by a stable period when cell death rate becomes equal to growth rate. At the exponential growth stage, a large amount of nitrate and ammonia is absorbed, due to the rapid proliferation of cells, for the synthesis of nitrogenous living substances. Accordingly, a rapid pH increase and decline is observed in nitrate and ammonia treated experiments, respectively. However, the growth and death rate of the cell reaches a steady state when the space and nutrients in the media have been utilized to the maximal potential. Consequently, the biomass growth shifts into a stable period, leading little additional changes in the pH.

A quick glance of the stoichiometry of Equation (5) reveals that the amount of carbon trapped in biomass (106 CO_2_) is significantly greater than that by alkalinity (16, assuming complete CO_2_ dissolution). The laboratory measurements (comparison of total carbon increase in the culture experiments to the added DIC, associated with pH decline from ~10.2 to ~8.4 after filtering out the biomass) were consistent with the theoretical implication.

It’s worth noting that the absence of N in the system still led to microbial growth (presumably through BNF), but the rate was sluggish and pH (7.5–8.5) little changed (Figure 1B). Furthermore, as predicted by Equation (6), the culture media under the ammonia condition exhibited a strong trend of acidity increase over time, where the pH, after an early increase from 7–7.5 in the first 3–5 days, experienced a steady decline, and ultimately reached ~4.5 at the end of the 20-day experiments (Figure 1B). The respective increase and decrease of pH associated nitrate and ammonia assimilation observed in the experiments are in total agreement with the proposition given by [15].

Organisms in general prefer ammonia over nitrate because of the lower energy cost for biosynthesis. If ammonia is absent and nitrate is the sole nitrogen source, cell growth usually occurs at a slower rate. For phytoplankton, the subdued growth leads to ~12% less biomass yield per unit N source (see summary by Raven et al. [16]). One way to compensate phototrophs for the excess energy consumption associated with nitrate assimilation may be stronger photosynthetic photon flux densities (PPFD). In field settings of shallow (<5 m) estuary, Møhlenberg [17] observed positive phytoplankton biomass growth at a minimal photon flux density of 0.2 mol m^−2^ d^−1^ and a saturation density of ~3 mol m^−2^ d^−1^ and ~15 mol m^−2^ d^−1^ in spring and summer, respectively. The illuminance of the fluorescent light used in the present study was 2000 lux, equivalent to ~2 mol m^−2^ d^−1^, significantly lower than the average saturation density reported by Møhlenberg [17], suggesting that the measured OH^−^ production can be further enhanced. Estimated PPFD at mid-latitude (37~56° north) is on the order of 43–48 mol m^−2^ d^−1^ [18], at least 300% stronger than the reported saturation density for phytoplankton growth, and certainly has sufficient intensity to be harvested to offset the nitrate disadvantage. Even at the yearly minimum (winter solstice), theoretically estimated PPFD at the latitude of 55° N comes to be approximately 5 mol m^−2^ d^−1^ [19] and above the ~3 mol m^−2^ d^−1^ saturation density for phytoplankton.

### 3.2. Carbonate Mineralization

When pH rises beyond ~8.92 in marine environment, Equation (3) can go through further disassociation via

**Figure 4:**
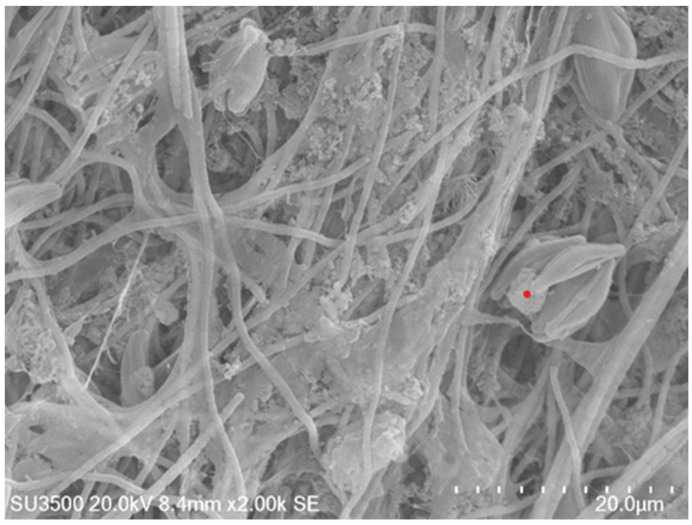
Scanning electron microscope (SEM) microphotograph of algae biofilm showing the formation of mineral phases on the cell surfaces. A typical composition of the inorganic precipitates (given by Energy-dispersive X-ray spectroscopy (EDX) analysis performed on the grain indicted by the red dot) is C (40.38% ± 4.01), O (47.2% ± 4.13), Mg (6.27% ± 0.21), and Si (6.15% ± 0.17), similar to the mineral phase (i.e., MgCO_3_ and SiO_2_).

HCO_3_^−^ + OH^−^ ⇔ CO_3_^2−^ + H_2_O (7)

to render the system dominated by carbonate anions. Given the 400 ppm (10^−2^ mol) and 1300 ppm (10^−1.27^ mol) average concentrations for Ca^2+^ and Mg^2+^ in seawater, it follows that the precipitation of calcite (CaCO_3_, solubility product K_sp_ = 10^−8.48^) and magnesite (MgCO_3_, K_sp_ = 10^−8.03^) becomes practically relevant to permanently storing the trapped CO_2_ into mineral forms, if Equation (7) yields a modest amount of CO_3_^2−^ (>10^−5^~10^−4^ mol). Speciation calculation via the thermodynamic code of PhreeQC [20] revealed the presence of 10^−3^~10^−2^ mol CO_3_^2−^ in the media at pH 10, corresponding to a saturation index (defined as the ratio of ionic activity product to K_sp_) of Ω = 2 and 1.27 for calcite and magnesite in seawater, respectively. This supersaturated condition was confirmed by the observed (Figure 4) extensive mineralization of calcite and amorphous magnesium carbonate phases on the biofilm. The absence of magnesite is expected because anhydrous magnesium carbonate has not been shown to crystallize at room temperature.

### 3.3. CO_2_ Trapping Methodology

Based upon the experimental testing results, we propose a microbially induced direct air capture (MI-DAC) strategy. The core concept is to increase seawater carbonate alkalinity without invoking mineral weathering. This approach relies on coastal marine phytoplankton farms to elevate seawater pH through microbial nitrate assimilation. The produced high pH water can be circulated back to ocean after aeration processes to absorb CO_2_. Unlike algae farms intended for biofuel production where specially selected phytoplankton rich in triacylglycerols is raised to maximize oil yield, MI-DAC can be executed using the entire marine photoautotrophic microbial community, eliminating the need to guard the culture chambers from contamination by unwanted microorganisms.

The intended strategy can be viewed as a coupled nitrate reduction-CO_2_ absorption process. On the surface, CO_2_ trapping occurs through biomass growth and hydroxyl alkalinity production. This is apparent as the assimilation of each nitrate ion consumes 6.625 CO_2_ molecules (the Redfield stoichiometry) via photosynthesis [13], while producing one OH^−^ to dissolve CO_2_. However, it is important to note that MI-DAC requires a steady input of nitrate to maintain phytoplankton growth. A straightforward calculation using the stoichiometry in Equation (5) shows a nitrate (in the form of nitric acid) consumption rate of ~187 Mt per Gt CO_2_ trapped. This parity ratio, if not amended, would put a severe constraint on the scales of MI-DIC operation, given the total size of 230 Mt for annual industrial nitrogen fixation (2018 data). To circumvent this restriction, we further propose an on-site nitrate recycling facility that converts organic N and ammonia from biomass to NO_3_^−^. This sets MI-DAC apart from conventional biological CO_2_ trapping methods such as photosequestration [21], biofertilizer manufacturing [22], and BECCS in that the resultant biomass (~0.76 ton wet or ~0.35 ton dry for every ton of CO_2_ trapped) is now considered a nitrogen holder and a transitory carbon storage. The new strategy instead focuses on taking advantage of the biologically spawned hydroxyl to absorb CO_2_ molecules through dissolution reactions (Equation (3)) and store it as carbonate alkalinity in seawater.

The regeneration of nitrate from ammonia can be fulfilled either through industrial processes or biological nitrification. Although the traditional chemical engineering method via the Haber-Bosch process is a mature technology and can perform with high (97%) yield, it is energy intensive due to the requirement of high temperatures (400~500 °C) and pressure (15~25MPa). On the other hand, microbial nitrification occurs at ambient conditions extensively and is used commonly in wastewater (including saline water) treatment. Biological nitrification technologies most relevant to MI-DIC may be akin to that used in aerobic tanks outfitted with moving-bed biofilm reactors (MBBR) for wastewater treatment [23]. Traditionally, the active bacteria in these settings are those that obtain energy by oxidizing ammonia (NH_3_ and NH_4_^+^, whose relative quantity depends on pH and salinity) to nitrate [24]. While the conventional view holds that biological nitrification is a two-step aerobic process carried out first by ammonia oxidizing bacteria (AOB, converting ammonium to nitrite) followed by nitrite oxidizing bacteria (NOB, further oxidation of nitrite to nitrate), new studies [25,26] found that certain *Nitrospira* bacteria are able to mediate the Complete Ammonia Oxidation (dubbed Comammox) process. Furthermore, recent metagenomic data from recirculating aquaculture systems (a process similar to nitrifying moving bed biofilm reactor (MBBR)) revealed additional diversity in the ammonia-oxidizing microbiological community, implying the potential resilience of nitrification function under a range of environmental conditions [27].

Additional factors external to MI-DIC, such as the carbon holding capacity of ocean and environmental consequence of seawater alkalinity increase, need to be evaluated to ensure the validity and safety of this strategy. The ocean has already absorbed ~40% of all anthropogenic C emissions, but on the whole, is still a carbon sink at present [28]. Global carbon cycle model suggests that the CO_2_ storage tonnage in the ocean is on the order of trillions, similarly to that underground. Furthermore, it is concluded that such capacity will allow the majority of the present atmospheric CO_2_, excessive to pre-industrial levels, to be absorbed naturally by the ocean [29], in a longer term (100 to 200 ka pending on estimated CO_2_ uptake rate). Collectively, current understanding appears to converge on the adequacy of ocean carbon sequestration capacity. For the alkalinity effect on ocean chemistry, consensus seems to remain in the making (at least for short term impact), as most existing work so far has focused on exploring the potential outcome of acidifying conditions. Nevertheless, assuming all the anthropogenic CO_2_ in the next 100 years is added to the ocean in the form of HCO_3_^−^ and CO_3_^2−^, the calculation showed that seawater alkalinity is not anticipated to greatly exceed 3 mEq/Kg [30]. While this represents a ~30% increase from today’s average value of ~2.3 mEq/Kg [31], it needs to be evaluated in conjunction with the ocean surface acidity that has experienced 0.1 pH unit decrease, due to atmospheric CO_2_ elevation. Moreover, available evidence suggests that about 17% of the added alkalinity can be used by coral reefs, leading to a ~7% increase in the net community calcification, as ocean chemistry is restored closer to pre-industrial conditions [32].

The MI-DAC process is a *de facto* coupled C and N biogeochemical cycle and can take advantage of anthropogenically elevated N presence in natural waters. Nitrogen pollution from fertilizers, livestock effluent, and fossil fuel combustion occurs widely in global aquatic systems. Artificially fixed nitrogen in seawater exists mostly in the form of nitrate [33] and can potentially facilitate the MI-DAC operation. This could be particularly fitting to major nitrogen fertilizing regions such as the east Asian sea, where anthropogenic NO_x_ emission has experienced more than a twofold increase since 2000 [34], along with a burst (1463 Gg N/year) of mostly nitrate depositions [35].

### 3.4. Cost Analysis

Cost has always been a highly contested issue for carbon reduction technologies, largely in view of financial sustainability. For DAC, an economic analysis conducted 10 years ago [36] gave an estimate of $250–$1200/tCO_2_. This number was further refined with consideration of thermodynamic efficiency and capital expenditure, and finally converged at ~$1000/tCO_2_ [8]. It was subsequently concluded that the cost was prohibitively high for DAC to be a climate mitigation tool. However, a later study [37] argued that the expense can be reduced to $309/tCO_2_ with optimized energy consumption and low-cost packing materials for the air-absorbent contacting device (contactor). A most recent estimation, based upon refined engineering design and the recyclability of the chemical absorbents, further lowered the cost to $94 to $232/tCO_2_ [38]. Despite the significant decline, current pricing is still far above the threshold of $25–30 range at which carbon capture and storage (CCS) systems can be deployed at a significant level [39].

Existing DAC technologies rely on CO_2_ absorption by alkaline substances of either liquid or solid state. Whereas CO_2_ trapping takes place at ambient conditions (with the help of air blowers), a complete operation needs to embed in a very energy and cost intensive process for CO_2_ liberation, to regenerate/recycle the sorbent. For this type of setup, strong heating (as high as 900 °C) is used to decompose precipitated carbonate salts (normally CaCO_3_) so that the original alkalinity (usually in the form of alkali metal hydroxide) can be recovered [40]. The use of solid sorbents lowers the CO_2_ desorption temperature but still requires heat at 80 to 480 °C [41]. The MI-DAC approach differs principledly from the existing ones in that CO_2_ discharge from sorbent (seawater in our case) is no longer part of the scheme. The projected process flow of MI-DAC is in fact similar to that of membrane bioreactor wastewater treatment plants (MB-WWTP), less the conventional components needed for solid waste separation and processing (such as sedimentation tank and sludge dehydration system). Accordingly, a cost analysis (hypothetical due to lack of constraints by field testing data) can be performed using a typical MB-WWTP as a template (assuming 1 Mt water/day processing capacity, Table 1). Investment to be considered for fixed assets (C_AS_) may include expenses for infrastructure construction, pump station, distribution well system, biofilm reaction tank, fan room, electrical controller, and other ancillary equipment. Operation & maintenance costs (C_OM_) may include items such as energy, microbial growth maintenance, water test, and labor. Assuming the levelized cost per ton CO_2_ captured from the air is the sum of the levelized C_AS_ and the annual C_OM_, our estimate gives a value of $30 to 31/tCO_2_ for a MI-DAC plant with a 30-year lifetime (Table 2). This low-cost stems to a large extent from the exclusion of absorbent regeneration/recycle and solid waste disposal operation that otherwise requires heavy capital and energy investment. Due to the lower costs of fixed assets, the MI-DAC approach run at ~10.5% of the C_OM_ to C_AS_ ratio, about 2.6 times greater than the calculated values for traditional DAC facilities [42], and over 0.5 times higher than that suggested for wastewater treatment plants.

More theoretical cost analyses of MB-WWTP operation intended for MI-DAC can be carried out using known cost functions. A straightforward thermodynamic calculation shows 0.13 moles of CO_2_ will be absorbed per liter of water to bring the pH down from 10.2 to 8.2. Assuming 80% of this rate is applicable to MI-DAC in seawater, a 1 Mt CO_2_/year MI-DAC plant requires a daily water processing capacity of ~630,000 m^3^. The full cost of a MB-WWTP at this scale, estimated via the published [43] cost function C(€) = 17.3617 V^0.5771^ *e*^(0.1006A + 0.6932COD)^ (V = volume of water/year, A = lifespan of the plant, COD = chemical oxygen demand removal efficiency for bacterial bed reactor), ranges from €47 to €24 for COD = 100% and zero, respectively. The chemical oxygen removal demand requirement for MI-DAC operation is minimal, in theory. Assuming 30% COD is enforced, the cost function given is a value of ~€29/tCO_2_, or slightly below $32/tCO_2_, based upon the conversion rate of €1 = $1.10, similar to the hypothetical estimation of $30–31/tCO_2._

The operations and maintenance (O&M) costs are estimated based on current industrial wastewater treatment plant (WWTP) operation with similar equipment and process. Energy costs were calculated using a commercial charge of $0.12/kW·h. Equipment maintenance cost accounts for 5% of total equipment cost. Other costs followed the average market-set price.

To complete the cost analysis, an on-site biological nitrification tank, intended to regenerate NO_3_^−^ from ammonia, is further budgeted in. Microbial removal of N from wastewater conventionally requires two separate operations: aerobic nitrification (oxidizing NH_4_^+^/NH_3_ to NO_3_^−^) and anaerobic denitrification (reducing NO_3_^−^ to N_2_). The aerobic process alone is sufficient for the MI-DAC purpose, as nitrate instead of dinitrogen is the intended product. Accordingly, the cost is expected to be a fraction of that for regular MBBR, due to the omission of anaerobic denitrification. The scale of the needed aeration facility depends on the surface area of the biofilm carrier and the nitrification rate, which in turn is a function of inlet ammonia loading rate (or hydraulic retention time) and dissolved oxygen content. The majority of existing MBBR find applications at relatively low nitrogen concentrations where the influent ammonia varies from <40 mg/L in municipal sewage [44] to ~500 mg/L in industrial wastewater [45], with the nitrification efficiency frequently reaching 80% and above. Within the range of 183–438 mg/L, NH_3_-N [24] observed a linear relation of rate_nitrification_ = (0.51r_inlet-ammonium_ + 0.53) and reported a nitrification rate of 3.05 gNH_4_/day/m^2^_biofilm_, when the inlet ammonium loading was maximized at 5 g m^−2^ day^−1^. At this rate, recycling one ton N will require ~420 × 10^3^ m^2^ of biofilm surface area. Using commercially available MBBR media (e.g., AlgaeControl Canada, Ecologix Environmental Systems) that offer >5000 m^2^/m^3^ active surface area, one estimates the footprint of such tanks will be ~84 m^3^.

For decomposed biomass where the (NH_3_ + NH_4_^+^) level can reach 5000 mg/L [46], the ammonia loading rate may be further increased by several folds, leading to potentially much more rapid nitrification, assuming the Torkaman et al. linear relation holds. Nevertheless, cost assessment for aerobic nitrification at such a high ammonia concentration is difficult to be specific without kinetic data. At present, we assume aerobic nitrification is 50% of a regular moving-bed biofilm reactor, whose expense can be estimated by the same cost function [43] of MB-WWTP, less COD removal. For a nitrate self-sufficient MI-DAC setup, 1 Mt CO_2_/year capacity needs a companion nitrification tank of ~300,000 m^3^ water/day, assuming an influent ammonia concentration of 3725 mg/L, or 75% of the (NH_3_ + NH_4_^+^) level reported for bio-waste [47]. The inclusion of such facility will lead to an additional $8.5 increase per ton CO_2_ captured, making the final cost to be around $40/tCO_2_. This value is drastically lower than any of the known DAC technologies have offered thus far (by a factor of at least 2.5~5.5, see review in [36] and Table 3), and is in fact approaching the number of $25–30/tCO_2_ suggested by most energy and economic modelling for the rapid deployment of large-scale CCS systems [37]. To this end, the MI-DAC strategy appears to hold great promise as a carbon management tool, given the 100~1000 ka residence time for alkalinity in ocean.

## 4. Conclusions and Outlook

Bench-top experiments confirmed that phytoplankton nitrate assimilation combined with CO_2_ dissolution can potentially be a low-cost DAC strategy for carbon mitigation. This method utilizes the hydroxyl alkalinity produced during microbial transformation of nitrate to ammonia to increase water pH. Laboratory tests showed that strong alkaline conditions of pH > 10 can be achieved in seawater. At this pH level, atmospheric CO_2_ is readily absorbed and converted to carbonate alkalinity, and in the long-term captured by carbonate mineralization in ocean. Nitrate can be recycled on-site through nitrifying biofilter from the harvested biomass, a by-product that can also be used as stock material for algal products such as green fertilizers.

A cost analysis based upon laboratory data indicated the MI-DAC approach has a strong likelihood to sharply cut down the expenses of carbon sequestration. The estimated cost of ~$40/tCO_2_ is merely ~20–40% of those offered by currently available technologies, and appears to suggest that this method at least has the potential to be another climate mitigation tool that meets the economic demands for large-scale deployment.

It is suggested [46] that, to meet the 1.5 °C target, atmospheric CO_2_ capture needs to operate at 10 Gt CO_2_ yr^−1^ by mid-century and 20 Gt·yr^−1^ by the end of century. There are about half a dozen known NETs, including reforestation, BECCS, DAC, soil carbon, biochar, and enhanced weathering, that can remove CO_2_ from air. Thus, on average, DAC is preferred to have an ability to trap 1.5 to 2 Gt CO_2_ per year (15–20% of the proposed total CO_2_ removal), by mid-century. Industrial-scale direct air capture using currently available methods may take 20–30 years to reach this desired capacity. Incidentally, a forestry/soil-based approach appears to be ready for massive deployment, but is unlikely to achieve its full capability because of the fierce competition with agriculture for land usage. In contrast, the WWTP technology-based MI-DAC approach may be made deployable much earlier at large scales, thanks to the milder R&D needs and lower costs, while competing for little natural resources and leaving no legacy of CO_2_ storage.

To reach the natural CO_2_ consumption level of continental weathering (~0.5 Gt CO_2_ per annum, [46]), the daily water processing capacity of MI-DAC needs to reach ~239 million m^3^. Putting this into perspective, the combined WWTP capability of US and China alone is ~310 million m^3^/day, indicating the scale of MI-DAC operations ought to be fully manageable in practice. On the other hand, there are ~40,000 wastewater treatment systems in the US, Europe, and China alone, and the top 10 largest wastewater treatment facilities in the world have a combined processing capacity of only ~25 million m^3^/day [53], equivalent to 54 Mt CO_2_/year, if used for MI-DAC. Thus, scaling up MI-DAC may require substantial initial investment to set up multiple facilities equivalent to the largest wastewater treatment plants in the world.

## Figures and Tables

**Table 1:** Fix asset investment ($million) for a 10^6^ tCO_2_/year MI-DAC operation.

	MC	EC	LC	DEC	Explanation and Justification
infrastructure construction	27.10	0.87	3.42	31.39	Including site preparation, pipeline layout, building and other infrastructure construction.
pump station	1.69	2.40	0.72	4.81	32 elevator pumps (8 stand-by pumps, 125 kW/unit) to satisfy 1M ton water/day processing capacity.
pretreat pool	1.10	1.84	0.65	3.59	Pretreatment unit is used to remove debris and sediments.
distribution well system	0.87	0.13	0.17	1.17	Pump system controlling and distributing input water flow based upon the monitored water pH.
biofilm reaction tank	35.26	25.51	5.10	65.87	Plastic substrates are used for biofilm attachment and lamps are provided for photoautotrophic microbes.
pharmacy input control	0.73	1.87	0.48	3.08	Nutrient supply unit with feedback system to maintain optimal biofilm growth.
clean poll	10.39	16.63	7.36	34.38	Quality control (e.g., chemical oxygen demand (CODp) and N) of the treated water before recycled back to the ocean.
fan room	1.37	7.02	1.76	10.15	Aeration is provided to avoid anerobic microbe formation at the bottom of the reaction tank.
electrical controller	0	4.53	1.94	6.47	Estimation is based on a current WWTP with a capacity of 0.1 M ton water/day, assuming the cost is scalable by multiplying a factor of 10.
other costs	0	6.76	2.40	9.16	Miscellaneous costs (~11% of total DEC) and expenses covering site maintenance, furniture, operation training, and corresponding labor costs.
total direct engineering costs				170.07	
indirect engineering costs				29.81	Including plant design, consultant, field construction supervision, insurance, and office furnishing.
total engineering costs				199.88	
project reserve funds				20.15	10% of total engineering costs.
total project costs				220.03	

MC: materials cost for building materials such cement, bricks, and steel. EC: equipment cost. LC: on-site labor cost for construction and operation. DEC: direct engineering costs (sum of MC, EC, LC and OC). Total direct engineering costs are the sum of DEC of all individual modules. Total engineering costs: sum of total direct engineering costs and indirect engineering costs. Project reserve cost is set up for contingencies during construction and off-budget expenses. Cost estimates are derived from equipment vendors, standard engineering reference sources, and online databases.

**Table 2:** Annual operations and maintenance expense and levelized cost for per tCO_2_ captured from the air ($million/year).

Operation & Maintenance Costs							Average C_AS_	Total
Energy	Agent	Strain	Equipment Maintenance	Sludge Disposal	Water Monitoring	Labor		
4.54	7.56	2.85	3.40	1.87	0.14	2.85	7.33	30.54

**Table 3:** Cost comparison of current direct air capture technologies.

Technology	Capacity (tCO_2_/a)	Cost Reported ($/tCO_2_)	References
Heat temperature aqueous solution	280,000	339	Keith et al. (2006) [48]
	1,000,000	278	Socolow et al. (2011) [49]
	1,000,000	356	
	1,000,000	255–270	Mazzotti et al. (2013) [50]
	1,000,000	188	Keith et al. (2018) [38]
Low temperature aqueous solution	360,000	139	Fasihi et al. (2020) [42]
	3600	219	Roestenberg (2015) [51]
Moisture swing solid	365	130	Lackner (2009) [52]
MI-DAC	1,000,000	30.54	This work

## Data Availability

Some or all data and models that support the findings of this study are available from the corresponding author upon reasonable request.

## Supplementary Materials

The following supporting information can be downloaded at: https://www.mdpi.com/article/10.3390/ijerph20010550/s1. Supplementary File S1: ESAW medium and WC medium.
