# Supplementary material for: Direct Air Capture of CO2 through Carbonate Alkalinity Generated by Phytoplankton Nitrate Assimilation
Source: Int J Environ Res Public Health. 2022 Dec 29;20(1):550. doi: 10.3390/ijerph20010550 (PMC9820007; doi:10.3390/ijerph20010550)
Supplement: Supplementary file 1 [file ijerph-20-00550-s001.zip › ijerph-2058995-supplementary.pdf]

1. ESAW medium
   NaCl 363 mM/L, Na_2SO_4 25.0 mM/L, KCl 8.04 mM/L, $NaHCO_3$ 2.07 mM/L, KBr 725 µM/L, H_3BO_3 372 µM/L, NaF 65.7 µM/L, $MgCl_2 \cdot 6H_2O$ 41.2 mM/L, $CaCl_2 \cdot 2H_2O$ 9.14 mM/L, $SrCl_2 \cdot 6H_2O$ 82 µM/L, $NaNO_3$ 549 µM/L, $NaH_2PO_4 \cdot H_2O$ 21 µM/L, $Na_2SiO_3 \cdot 9H_2O$ 105 µM/L, $FeCl_3 \cdot 6H_2O$ 6.56 µM/L, $Na_2EDTA \cdot 2H_2O$ 6.56 µM/L, $ZnSO_4 \cdot 7H_2O$ 254 nM/L, $CoSO_4 \cdot 7H_2O$ 5.69 nM/L, $MnSO_4 \cdot 4H_2O$ 2.42 µM/L, $Na_2MoO_4 \cdot 2H_2O$ 6.1 nM/L, Na_2SeO_3 1 nM/L, $NiCl_2 \cdot 6H_2O$ 6.3 nM/L, Thiamine-HCl 297 nM/L, Biotin 4.09 nM/L, B_{12} 1.47 nM/L

2. WC medium

   $NaNO_3$ 1mM/L, $CaCl_2 \cdot 2H_2O$ 0.25 mM/L, $MgSO_4 \cdot 7H_2O$ 0.15 mM/L, $NaHCO_3$ 0.15

   mM/L, $Na_2SiO_3 \cdot 9H_2O$ 0.1 mM/L, K_2HPO_4 0.05 mM/L, H_3BO_3 0.39 mM/L, trace elements: $Na_2EDTA \cdot 2H_2O$ 11.7 µM/L, $FeCl_3 \cdot 6H_2O$ 11.7 µM/L, $CuSO_4 \cdot 5H_2O$ 10 nM/L, $ZnSO_4 \cdot 7H_2O$ 76.5 nM/L, $CoCl_2 \cdot 6H_2O$ 42 nM/L, $MnCl_2 \cdot 4H_2O$ 910 nM/L, $Na_2MoO_4 \cdot 2H_2O$ 26 nM/L, Na_3VO_4 98 nM/L, Vitamine B_{12} 0.1 µM/L, Thiamine Vitamin 1.11 µM/L, Biotin Vitamine, 0.1 µM/L
